# HIV prevention: better choice for better coverage

**DOI:** 10.1002/jia2.25872

**Published:** 2022-01-14

**Authors:** Linda‐Gail Bekker, Carey Pike, Sharon L. Hillier

**Affiliations:** ^1^ The Desmond Tutu HIV Centre, Faculty of Health Sciences University of Cape Town Cape Town South Africa; ^2^ Department of Obstetrics, Gynaecology, and Reproductive Sciences University of Pittsburgh School of Medicine Pittsburgh Pennsylvania USA; ^3^ Magee‐Womens Research Institute University of Pittsburgh School of Medicine Pittsburgh Pennsylvania USA

**Keywords:** pre‐exposure prophylaxis, long‐acting PrEP, HIV prevention, oral PrEP, injectable PrEP, PrEP implants

## Abstract

**Introduction:**

Antiretroviral‐based pre‐exposure prophylaxis (PrEP) is today an established, effective and safe method of HIV prevention used in multiple countries worldwide by a broad range of populations at risk of HIV infection. Biomedical innovations are critical in supporting the primary prevention of HIV; however, their potential can only be maximized if end‐user challenges are recognized, described and used to develop next‐generation models.

**Discussion:**

First‐generation PrEP, a daily oral pill, is highly efficacious, discreet and affords users the ability to commence and conclude treatment rapidly. However, consistent daily adherence and persistence is challenging, especially among younger populations, due in part to side effects, the risk of stock‐outs and a lack of pill storage options. Second‐generation PrEP, longer acting agents that require less frequent dosing, could overcome such challenges. Agents that have shown efficacy in clinical trials include a monthly vaginal ring and PrEP injectables to be administered every 8 weeks, while products in development include 6 monthly injectables, oral therapy that uses monthly rather than daily pills, implants and the potential for long‐acting passive immunization.

**Conclusions:**

Second‐generation PrEP agents will have the potential to offer improved adherence and less frequent reminders once they have undergone further development and the delivery systems that will best support them have been established. In order to pursue global UNAIDS targets of reducing new HIV infections to fewer than 500,000 annually by 2025, and to ensure that all people have access to prevention options that meet their specific prevention needs, both early and next‐generation PrEP options are needed.

## 1 INTRODUCTION

We have known for more than a decade that antiretroviral‐based pre‐exposure prophylaxis (PrEP) provides robust protection against HIV infection in all populations and through all routes of infection [1, 2, 3]. Today, 1.3 million people have accessed this form of biomedical prevention, yet this is dismally short of the 3 million UNAIDS hoped would have done so by 2020. 1.7 million new HIV infections still occurred in 2020, a rate three‐fold higher than the targets set out by UNAIDS [4]. There is no doubt that primary prevention must remain a focus in our AIDS response and that PrEP is a key player. Prior to the introduction of PrEP, options for discreet, user‐controlled HIV prevention for sexually active people were limited. People were largely dependent on male latex condoms, sero‐sorting strategies and medical male circumcision.

First‐generation PrEP consists of daily oral tenofovir disoproxil fumarate with emtricitabine (TDF/FTC). When taken at the prescribed dose, this daily pill has been shown in randomized controlled trials (RCTs) to be efficacious [1, 2, 5, 6, 7, 8]. If adherence is high, then protection too is consistently high, but low adherence leads to a lack of efficacy. Additionally, pharmacological modelling studies suggest that HIV exposure via the vaginal mucosa may require daily PrEP dosing for protection, while when exposure occurs during receptive anal sex, fewer than daily doses are required. This fact has given rise to “on demand” or coitally linked PrEP: the so‐called 2‐1‐1 dosing strategy [9]. Effective particularly in men who have sex with men (MSM), this strategy is as protective as daily PrEP and is preferred by some users [10].

The next advancement occurred with novel antiretroviral (ARV) agents, namely tenofovir alafenamide (TAF). This nucleoside reverse transcriptase and pro‐drug is more easily absorbed, with consequently higher drug levels, improved pharmacokinetics and fewer side effects compared to TDF [11]. TAF in combination with emtricitabine was found to be as effective as F/TDF in MSM, with a study to confirm its use in cis‐gender women recently launched.

PrEP has already impacted the lives of many who may otherwise be at risk of HIV acquisition. Testimonies of how intimacy is once again possible without fear and stigma have been heard. When used in combination with universal test and treatment approaches, using an overall sero‐neutral approach, it has led to reductions in population‐level HIV incidence at regional and city levels [12, 13, 14, 15].

## 2 DISCUSSION

### 2.1 First‐generation PrEP agents

Despite its high level of effectiveness, daily oral PrEP is not a feasible option for everyone. In the POWER study, conducted among young women in Kenya and South Africa across a variety of PrEP distribution platforms, the PrEP user journey was probed to understand enablers and barriers to effective daily use. While oral PrEP is highly suitable for some, there are a number of contexts in which daily, oral pills can become barriers to early use and persistence and may lead to pauses or even discontinuation [16]. Across studies in different populations and region, despite initial enthusiasm, there is evidence of inadequate daily PrEP adherence [17]. The SEARCH study in Uganda and Kenya highlighted difficulties among young women and young men, and this has been confirmed in other studies involving youth and adolescents [12, 17, 18, 19].

Hosek and colleagues conducted some of the first PrEP studies among adolescent MSM at the same time as heterosexual, South African adolescents were being engaged, with similar results: reasonable adherence at the time of PrEP initiation but decreasing adherence over time [20, 21]. There is an additional challenge with persistence. This pattern of remarkable uptake among young women at the start, followed by a short drop off in both persistence and return after 1–3 months, has also been observed among MSM and transgender women (TGW), where in one study, one fifth of individuals exited the programme at month 6, citing negative partner reactions and hostile services from providers as the main reasons for exit [22, 23]. There is, however, increasing evidence that this lack of persistence might not be permanent and that users may “cycle back” in and restart PrEP. This was well described in a study by Serota and colleagues among young MSM in the United States, where a complex stopping and restarting pattern occurred over time [24].

Ultimately, the first generation of daily, oral PrEP is here to stay, with positive features like high effectiveness in those who can use it, but negative features, which preclude its use by many people who need prevention products. The ability to stop and start and to use the on demand strategy for some people is important. The fact that PrEP can be sent in the post, by courier or delivered by peers is another key strength of this prevention tool, as is the ability to use it discreetly. On the other hand, the challenges of adherence and persistence, partially due to side effects, the risk of stock‐outs and reports that some people find the use of pillboxes and the storage of pills problematic must be acknowledged.

### 2.2 Challenges of adherence and persistence

This raises the socio‐behavioural question of why daily adherence to a prevention pill is so challenging for so many. Structural and emotional challenges aside, motivations do not always translate into health actions and behavioural economics teaches us that this is not unique to the field of HIV. Behavioural biases impact our daily decision making, reducing our ability sometimes to act consistently in our own best interests [25]. Exercise is a stereotypical example, where 74% of new users of health apps stop using them a mere 2 weeks from the time of the download. Fully half of those who commence an aerobic exercise programme stop within 6 months [26, 27]. A study on gym attendance in Brazil found that people were more likely to stay in an exercise programme if they were older and motivated by improving their physique and feeling healthy compared to those who were motivated solely by weight loss [28]. People may make the best decisions when they are provided with positive feedback, have an easy plan to follow and an unambiguous decision to make. Oral PrEP relies on daily good decision making, which is more challenging for some than others. PrEP demonstration projects have given some clue this may be more challenging for younger individuals and MSM may cope better than young women.

A solution to this could be less frequent dosing with alternative types of agents, specifically long‐acting formations. Insight gained from psychiatry where both oral and long‐acting injectable formulations of anti‐psychotic medications are available showed that long‐acting agents improved adherence and persistence, while additionally supporting early detection of non‐adherence compared to oral formulations, where non‐adherence often went undetected until major problems developed [29, 30].

Lessons can also be drawn from the reproductive health field and the use of long‐acting reversible contraceptions (LARCs). LARCs have gained popularity in recent years, especially among the youth and within sub‐Saharan Africa, due to the need for less frequent dosing, lower side effects and greater effectiveness [31, 32, 33]. The rates of contraception failure are significantly lower among LARC, and hormonal injectable users compared to those who rely on contraceptive pills, patches and rings. There is a similar reduction observed in the rates of unintended pregnancies, and this holds for young women [34]. In the “UChoose” study in South Africa that employed a cross‐over design, young women were randomized to receive contraceptive oral pills, rings and injectables. Participants were asked to imagine this was an HIV prevention modality and to correspondingly describe their preferences. The predominant reason against daily oral contraception, and hence daily oral PrEP, was that three quarters of participants feared they would forget to regularly take the tablets [35].

### 2.3 Second‐generation PrEP agents

**Figure 1:**
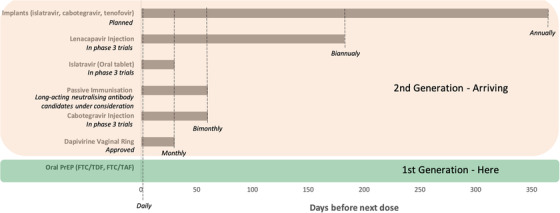
Dosing frequency for current and emerging HIV pre‐exposure prophylaxis options.

Second‐generation PrEP agents consist predominantly of longer acting agents that require less frequent dosing (Figure 1). The first such agent under consideration is the dapivirine (DVR) vaginal ring, which is a silicone ring infused with DVR, a non‐nucleoside reverse transcriptase inhibitor, which is inserted monthly into the vagina. The ring remains in‐situ until it is removed at the end of the month. A 90‐day ring has additionally been evaluated in a phase 1 study [36]. Two RCTs with the DVR ring indicated an overall efficacy of 30%, and effectiveness improved to 50% when the trial was converted to open label and adherence was improved [37, 38]. Adherence and thus effectiveness was shown to be better in older women. In a study which directly compared adherence to daily oral PrEP and the DVR vaginal ring among African adolescents aged 16–21, adherence to the ring was higher than to daily oral PrEP, suggesting that this option may be of benefit to adolescents who cannot use daily oral PrEP consistently [39]. The World Health Organization now recommends the DVR for second‐line HIV prevention in women in low‐ and middle‐income settings after daily oral PrEP. This is a real option for women, with the advantage of less frequent dosing, fewer side effects, potentially fewer health visits as it can be self‐inserted and an associated increase in discretion. However, the use of a vaginal ring does require practice to gain confidence in self‐insertion and correct placement. Multi‐purpose technologies (MPTs), which may combine HIV prevention and contraception progestins, are under development, but until they are approved, other sexual and reproductive health needs must also be considered.

Injectable PrEP options have shown significant promise in recent years. Cabotegravir, a strand transfer integrase inhibitor, is delivered in a long‐acting suspension via an intramuscular injection every 8 weeks. Two trials have shown superiority to daily, oral TDF/FTC when compared in double‐blind, double placebo studies. HPTN 083 was conducted among cis‐gender men and TGW who have sex with men and showed a lower HIV incidence in cabotegravir users versus TDF/FTC users (0.41 vs. 1.22 per 100 person years) [40]. HPTN 084 also reported a lower incidence of HIV among cis‐gender African women randomized to cabotegravir compared with TDF/FTC daily [41]. Notably, HIV incidence was lower than expected in both arms of the study, suggesting that women had also derived benefit from the oral PrEP arm [40, 41]. Long‐acting injectables certainly offer better adherence and may require less frequent reminding. On the other hand, there remain several unanswered questions around the implementations and use of injectable options. Clinically, it is unknown whether the residual drug following cessation of injections could contribute to selection for HIV variants resistant to integrase inhibitors. Logistically, it is unknown how accessible and feasible injectable options would be for those who migrate from their initiating clinic and additionally which healthcare professionals would be trained and approved to administer these intramuscular gluteal injections. Both HPTN 083 and 084 have moved into open label extension phases and will help to answer some of these questions, while it is hoped that demonstration and implementation studies will quickly address these knowledge gaps.

Concurrently, there have been advancements in the field of passive immunization. Results from the much‐anticipated antibody‐mediated prevention (AMP) trial were recently published [42]. The AMP study was conducted across multiple populations globally and sought to provide a proof‐of‐concept that a broadly neutralizing antibody, in this case VRCO1, infused intravenously every 8 weeks could prevent HIV. The study provided proof‐of‐concept that monoclonal antibodies can block HIV acquisition with the caveat that HIV infection prevention was dependent on viral neutralization sensitivity to the infused monoclonal antibody. VRCOI did not prevent the acquisition of isolates with an IC80 greater than 1.0 μg/ml and as only 30% of control arm acquired isolates had an IC80 less than this threshold, the overall prevention efficacy was 26.6% in HVTN/HPTN 704/085 and 8.8% for HVTN/HPTN 703/0.81 [42]. The AMP study gave the positive signal to further develop this prevention strategy but that improved and longer acting monoclonal antibodies are needed. The pipeline fortunately remains full of promising long‐acting neutralizing antibodies having a broader spectrum and higher potency, especially if the potential to administer a cocktail of bNAbs is realized [43].

Alongside these second‐generation strategies, which already offer a substantial evidence‐base, there are several further innovative products, which have entered clinical trials. This includes islatravir (ISL), a nucleoside reverse transcriptase translocation inhibitor, which is an exceptionally potent antiviral agent with a novel mode of action. ISL has been formulated as a tablet is taken once monthly. Blinded safety and pharmacokinetic data from the phase 2a trial have recently shown that pre‐specified efficacious thresholds were achieved at monthly doses of both 60 and 120 mg and an outstanding safety and tolerability profile [44]. ISL has moved into phase 3 trials, including Impower 022, where it is being tested among cis‐gender women in Africa and the United States, with TDF/FTC as a comparator. Impower 024 is evaluating efficacy among MSM and TGW who have sex with men compared to TDF/FTC or TAF/FTC. The study also employs a double‐blind, double placebo design and is end‐point driven. ISL is further being developed as an implant, based on the implantable contraceptive device containing etonogestrel, with the first pharmacokinetic study results released in 2019 [45]. This option offers the benefit of reversibility of removal, unlike the depot injectables and could last up to a year, it also has potential for MPT use. Two other implants were recently showcased in non‐human primate studies, namely a TAF implant and a cabotegravir reservoir implant, both showing promise [46, 47].

Another new agent under development as a very long‐acting ARV and PrEP agent is lenacapavir, a capsid inhibitor that is formulated to be delivered via subcutaneous injection every 6 months. This agent is under evaluation in phase 3 trials, first in young women and adolescent girls with a dual objective to study TAF/FTC and concurrently assess the safety and efficacy of lenacapavir in approximately 5000 cis‐gender women (ClinicalTrials.gov Identifier: NCT04994509). This large study will have two primary endpoints of comparing lenacapavir and F/TAF as an oral pill compared to background HIV incidence [48].

## 3 CONCLUSIONS

Overall, these first‐ and second‐generation PrEP for HIV prevention options represent the new array of “superheroes” of prevention. Each potent biomedical intervention must be delivered with a tailored package that includes access to counselling, condoms, contraception, lubricant, STI screening, and other sexual and reproductive health needs with additional harm reduction modalities tailored to the population in question. Second‐generation PrEP specifically offers the promise of improved adherence, less frequent reminders and potentially fewer clinic visits. Importantly, discretion is also an attribute for most of these second‐generation products. There is still much that needs to be understood about how these agents can be safely and effectively started and stopped and to determine the types of clinics, delivery systems, service models and providers that will be most effective and appropriate for efficient and accessible distribution to a variety of different populations.

As humans, we come in many different shapes and forms – options for HIV prevention need to match this variation. Preference may vary depending on timing, circumstances, life courses and relationships, and preference requires choice. The spaces where these choices are offered will need to, in turn, be differentiated and tailored to optimize their function, utility and scale‐up. Only when we can provide choice in such a manner can we expect to achieve better coverage of all people and better coverage of all HIV exposures.

## COMPETING INTERESTS

SLH reports receiving consulting honoraria from Merck, Gilead and ViiV and her institution receives funding from Merck. LGB reports receiving honoraria for advisory committee input to ViiV, Gilead, Merck (MSD) and Jansen. The DTHF has received grants from Gilead. CP has no competing of interests to declare.

## AUTHOR CONTRIBUTIONS

LGB, CP and SLH jointly conceptualized and developed this manuscript. LGB and CP compiled the first draft. LGB, CP and SLH all reviewed, edited and gave input into the manuscript. All authors have read and approved the final manuscript.
